# Supplementary figures and images for: Evaluation of Mitochondrial Function in Blood Samples Shows Distinct Patterns in Subjects with Thyroid Carcinoma from Those with Hyperplasia
Source: Int J Mol Sci. 2023 Mar 29;24(7):6453. doi: 10.3390/ijms24076453 (PMC10094811; doi:10.3390/ijms24076453)

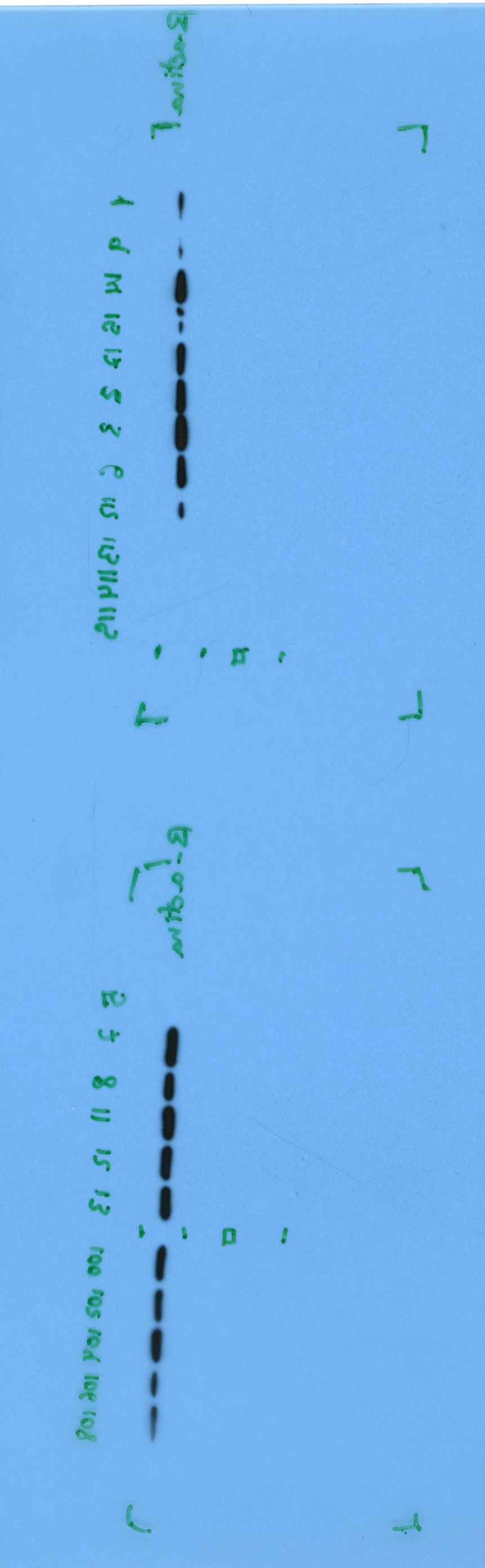

Supplement: Supplementary file 1 [file ijms-24-06453-s001.zip › Data + WBs/WBs/Thyroid tissue/Fig. 2C/WB-b-act (Fig. 2C).tif]

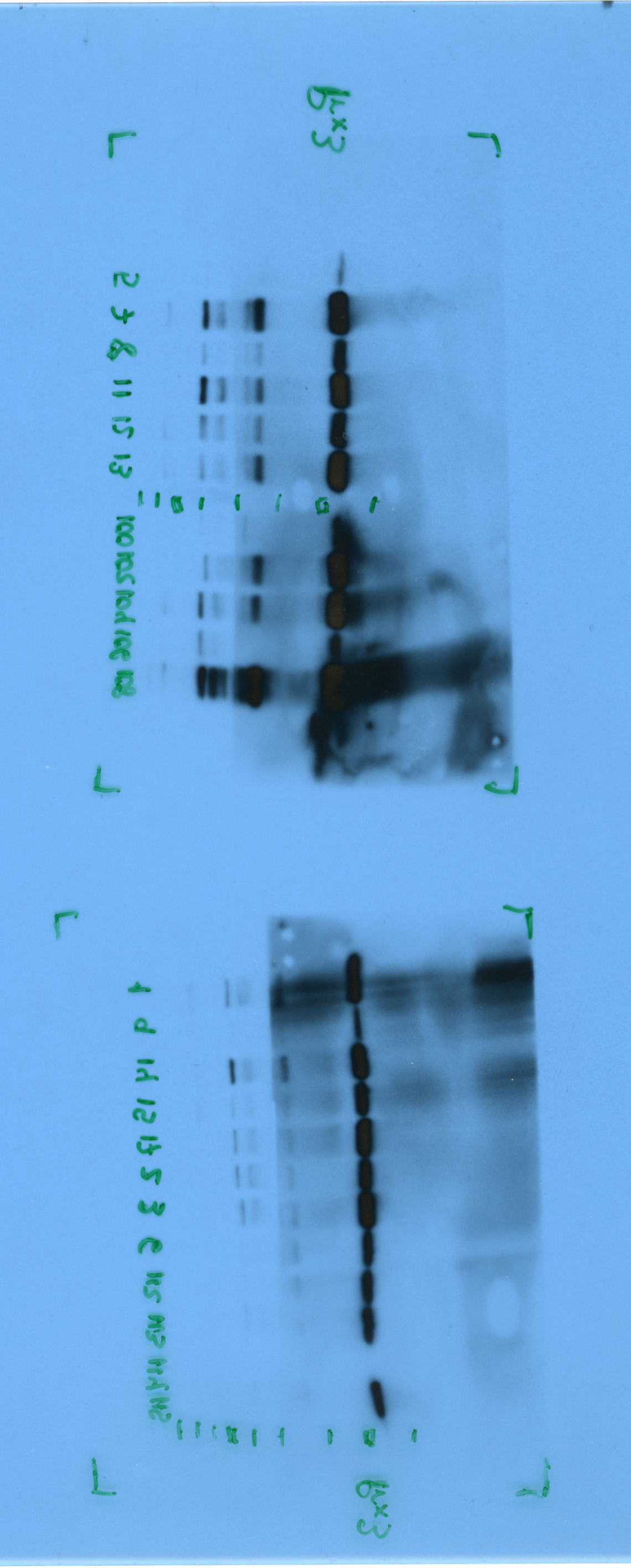

Supplement: Supplementary file 1 [file ijms-24-06453-s001.zip › Data + WBs/WBs/Thyroid tissue/Fig. 2C/WB-Prx3 (Fig. 2C).tif]

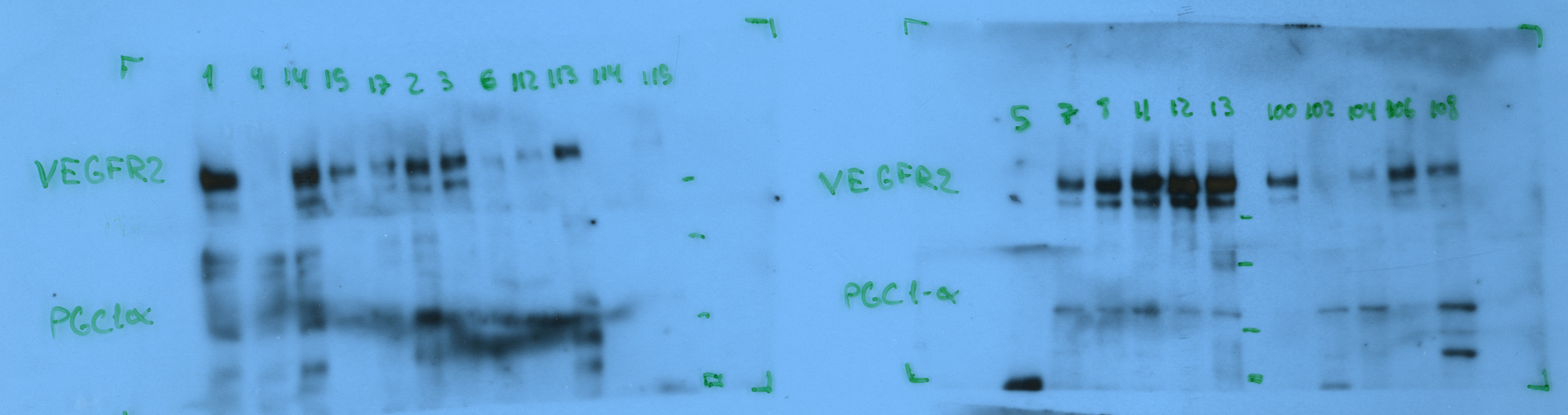

Supplement: Supplementary file 1 [file ijms-24-06453-s001.zip › Data + WBs/WBs/Thyroid tissue/Supp. Fig. 1/VEGFR2 (Supp. Fig 1)a.tif]

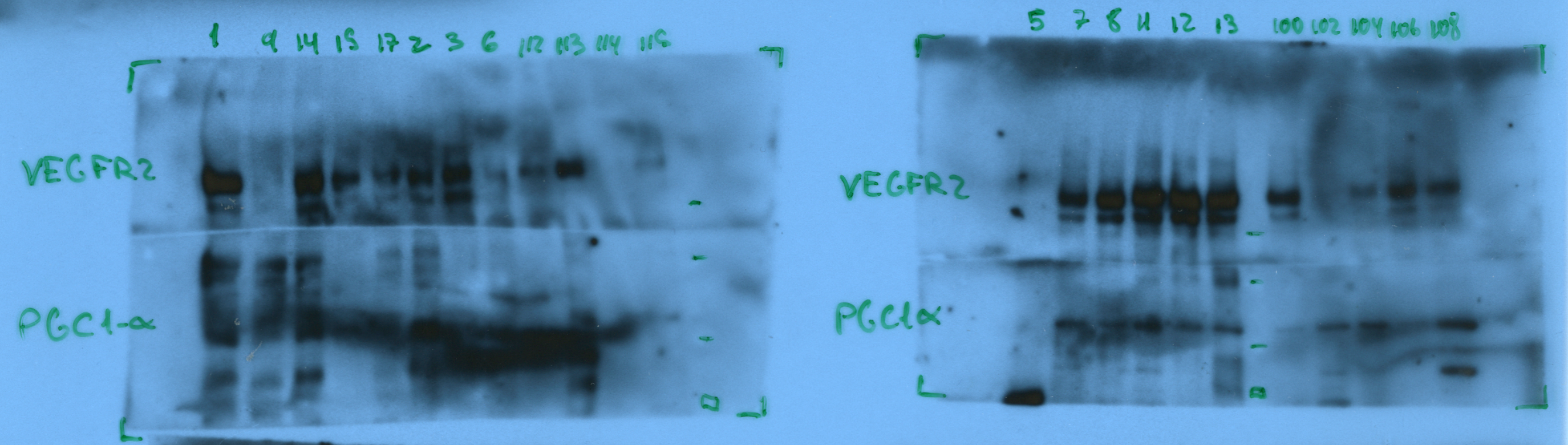

Supplement: Supplementary file 1 [file ijms-24-06453-s001.zip › Data + WBs/WBs/Thyroid tissue/Supp. Fig. 1/VEGFR2 (Supp. Fig. 1)b.tif]

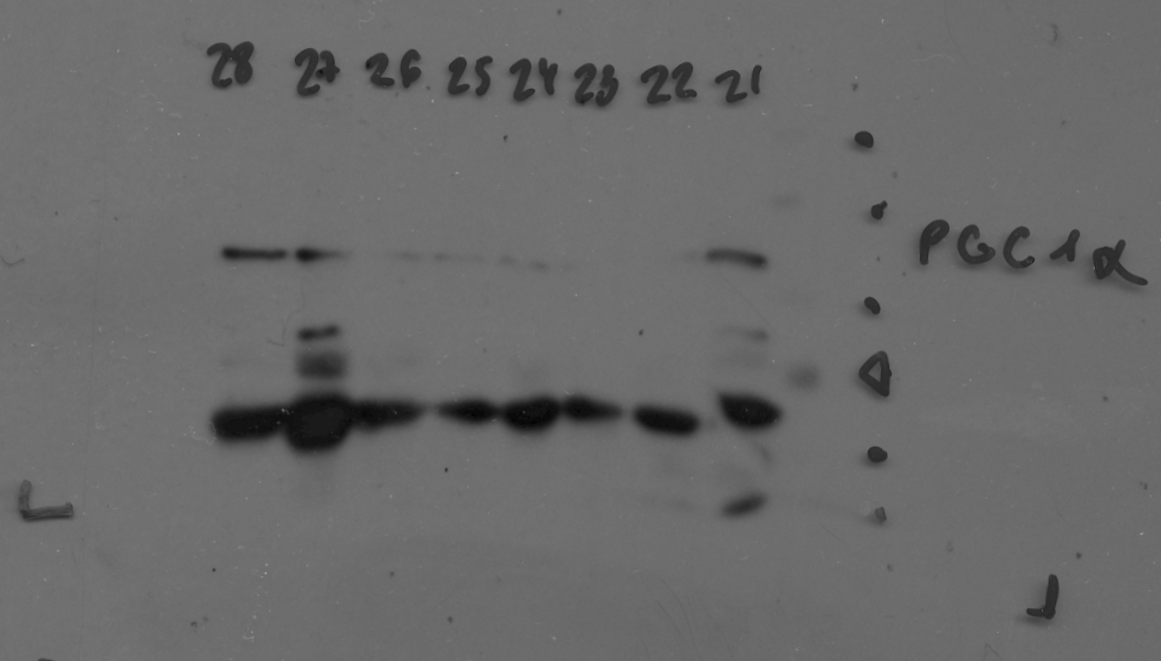

Supplement: Supplementary file 1 [file ijms-24-06453-s001.zip › Data + WBs/WBs/PBMCs/Fig. 3A/WB PGC-1a/WB-PGC-1a:3.tiff]

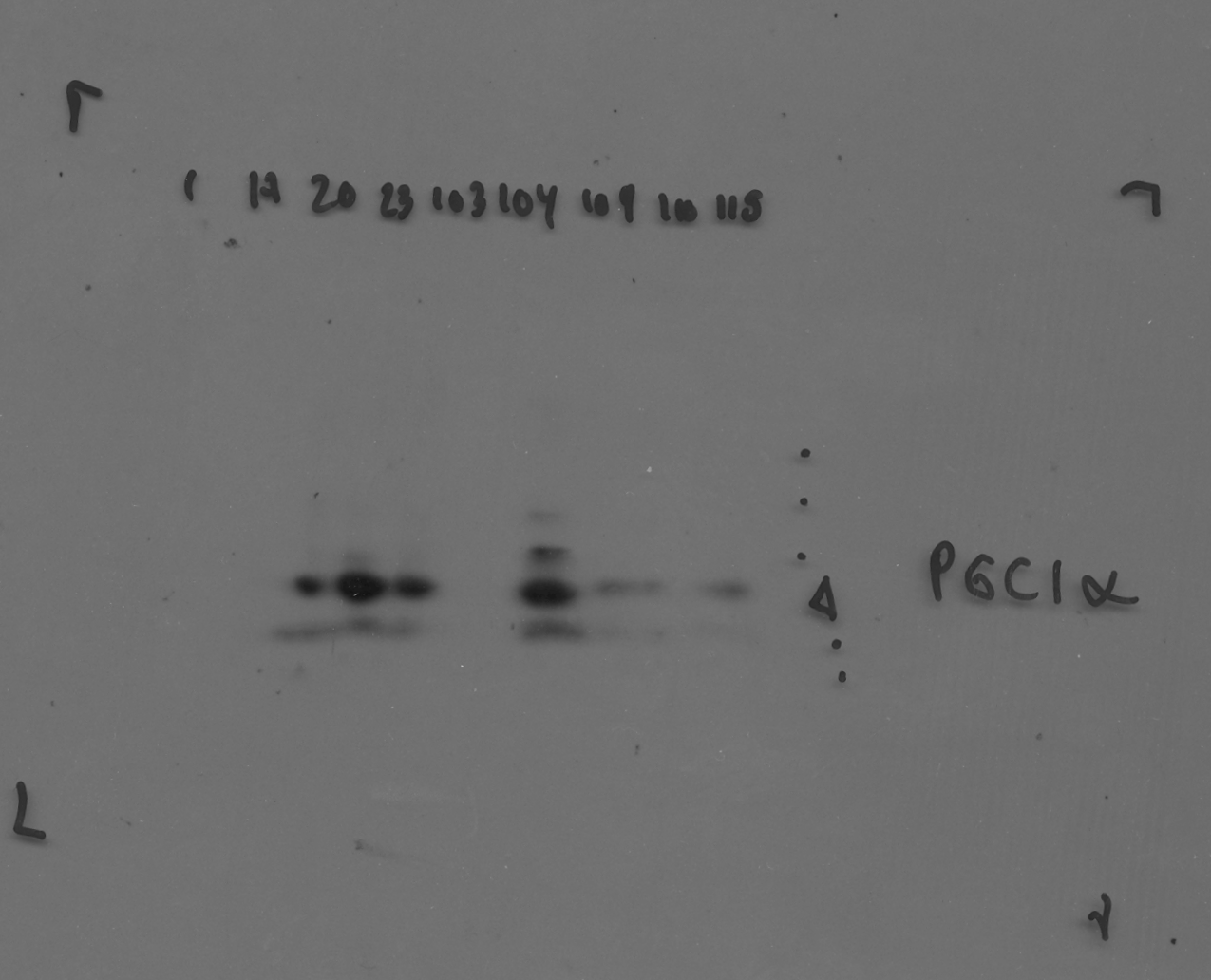

Supplement: Supplementary file 1 [file ijms-24-06453-s001.zip › Data + WBs/WBs/PBMCs/Fig. 3A/WB PGC-1a/WB-PGC-1a:2b.tiff]

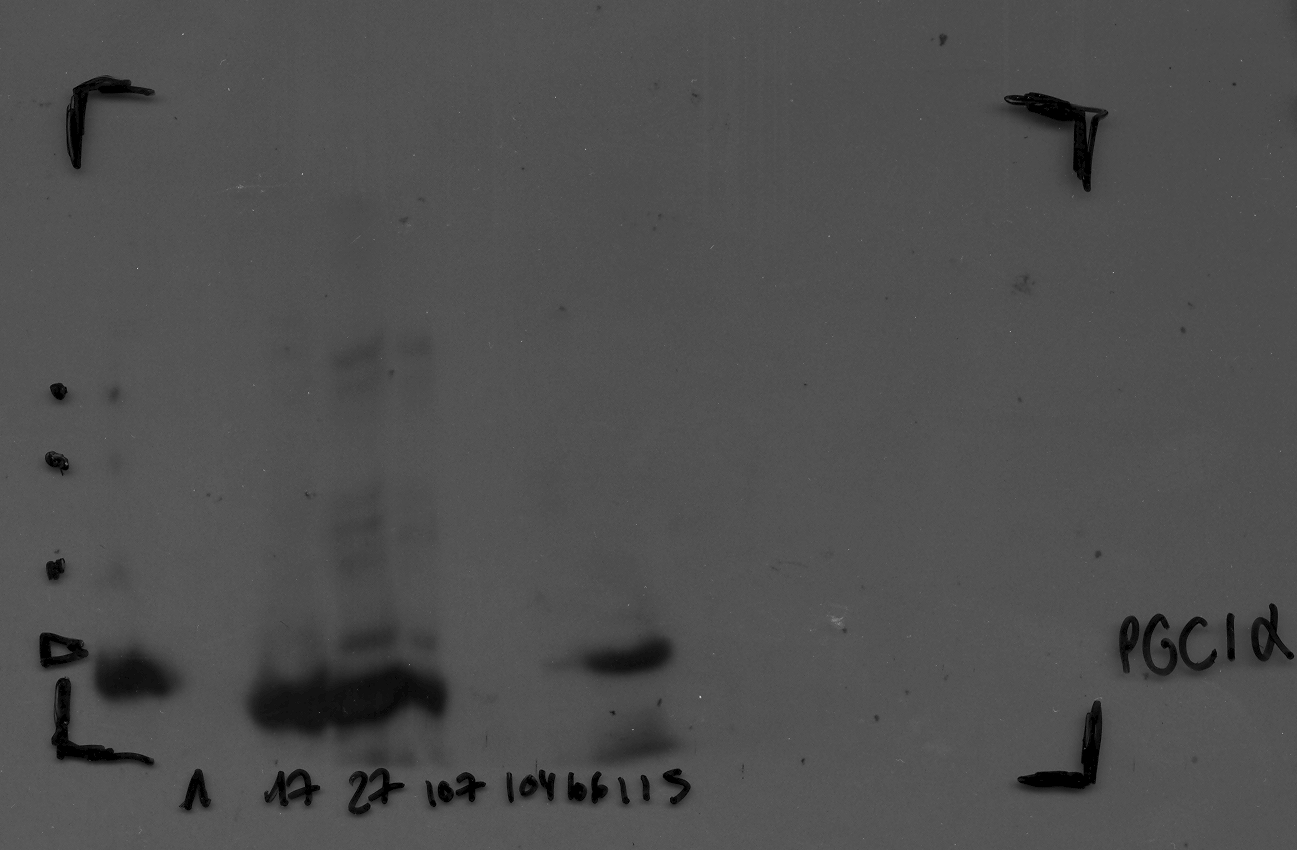

Supplement: Supplementary file 1 [file ijms-24-06453-s001.zip › Data + WBs/WBs/PBMCs/Fig. 3A/WB PGC-1a/WB-PGC-1a:2c.png]

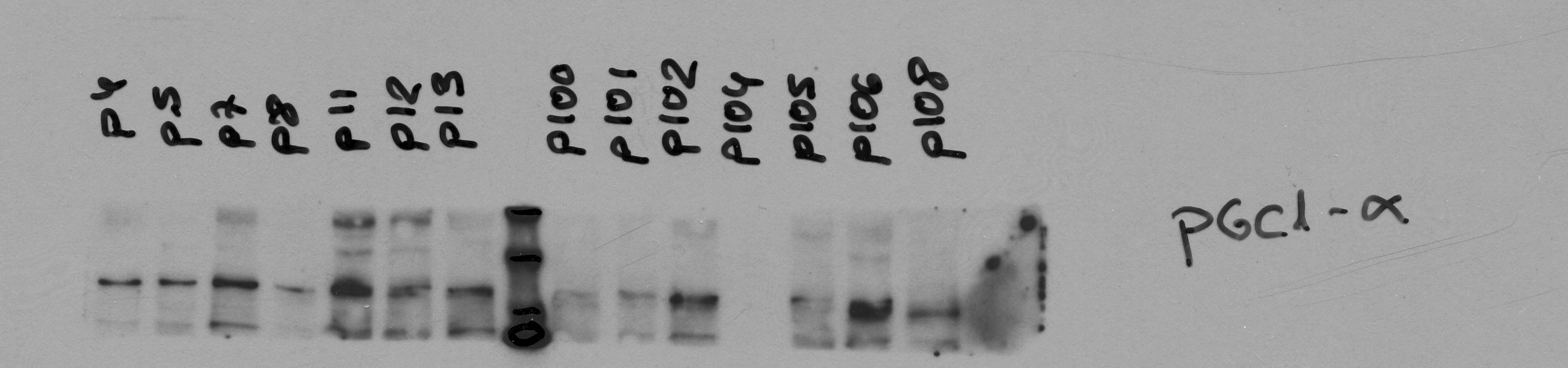

Supplement: Supplementary file 1 [file ijms-24-06453-s001.zip › Data + WBs/WBs/PBMCs/Fig. 3A/WB PGC-1a/WB-PGC-1a:4.tiff]

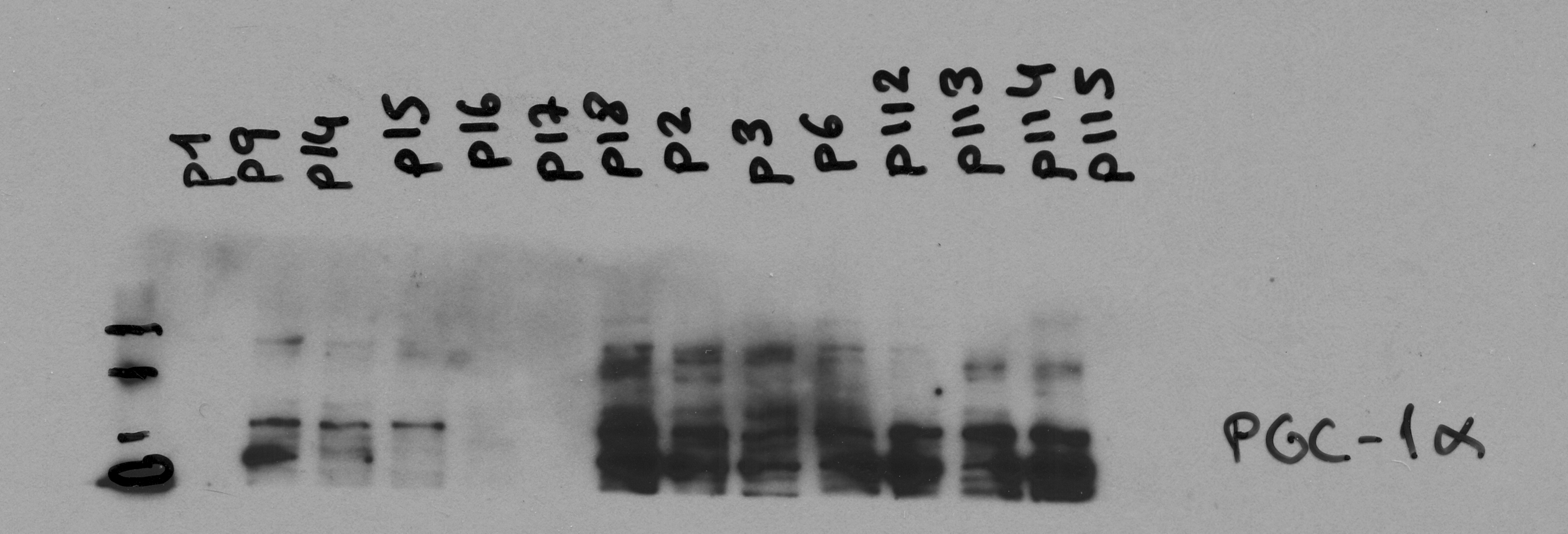

Supplement: Supplementary file 1 [file ijms-24-06453-s001.zip › Data + WBs/WBs/PBMCs/Fig. 3A/WB PGC-1a/WB-PGC-1a:5.tiff]

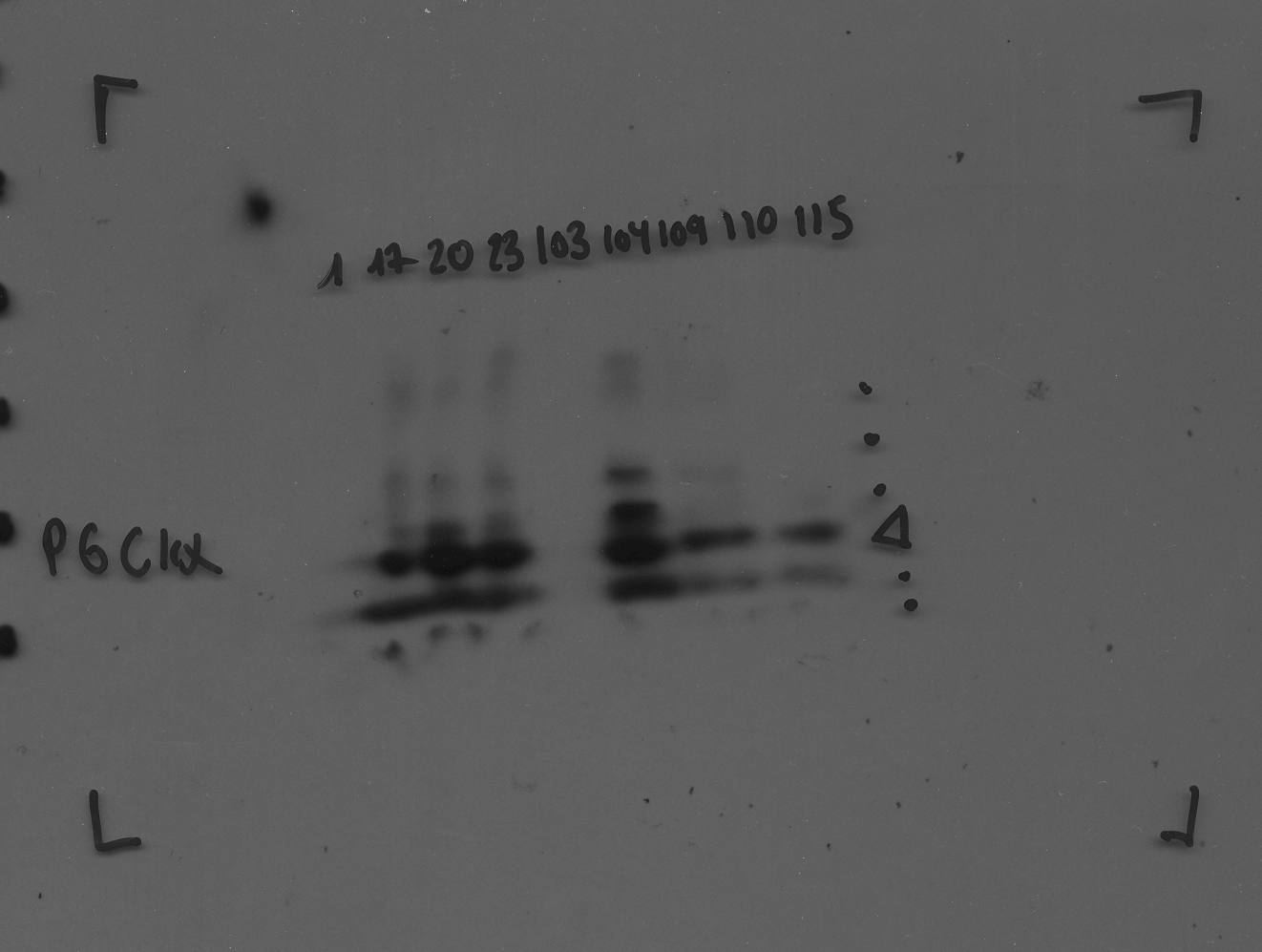

Supplement: Supplementary file 1 [file ijms-24-06453-s001.zip › Data + WBs/WBs/PBMCs/Fig. 3A/WB PGC-1a/WB-PGC-1a:2a.tiff]

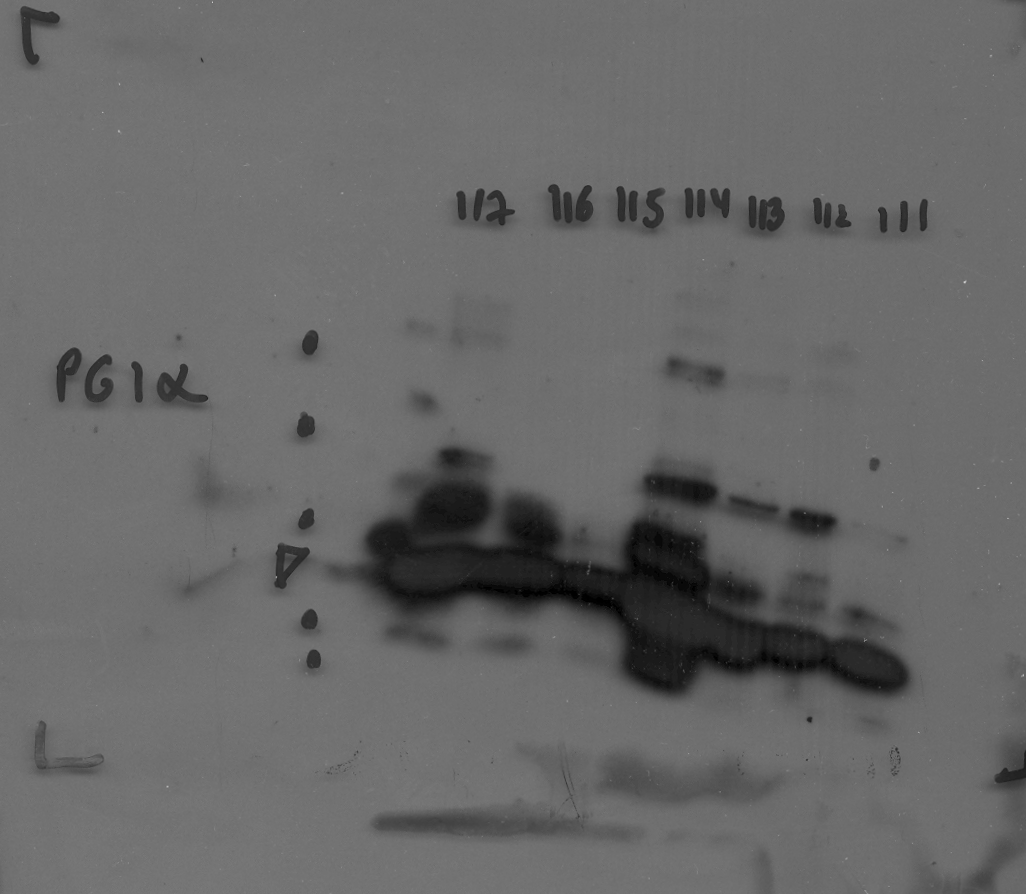

Supplement: Supplementary file 1 [file ijms-24-06453-s001.zip › Data + WBs/WBs/PBMCs/Fig. 3A/WB PGC-1a/WB-PGC-1a:1.tiff]

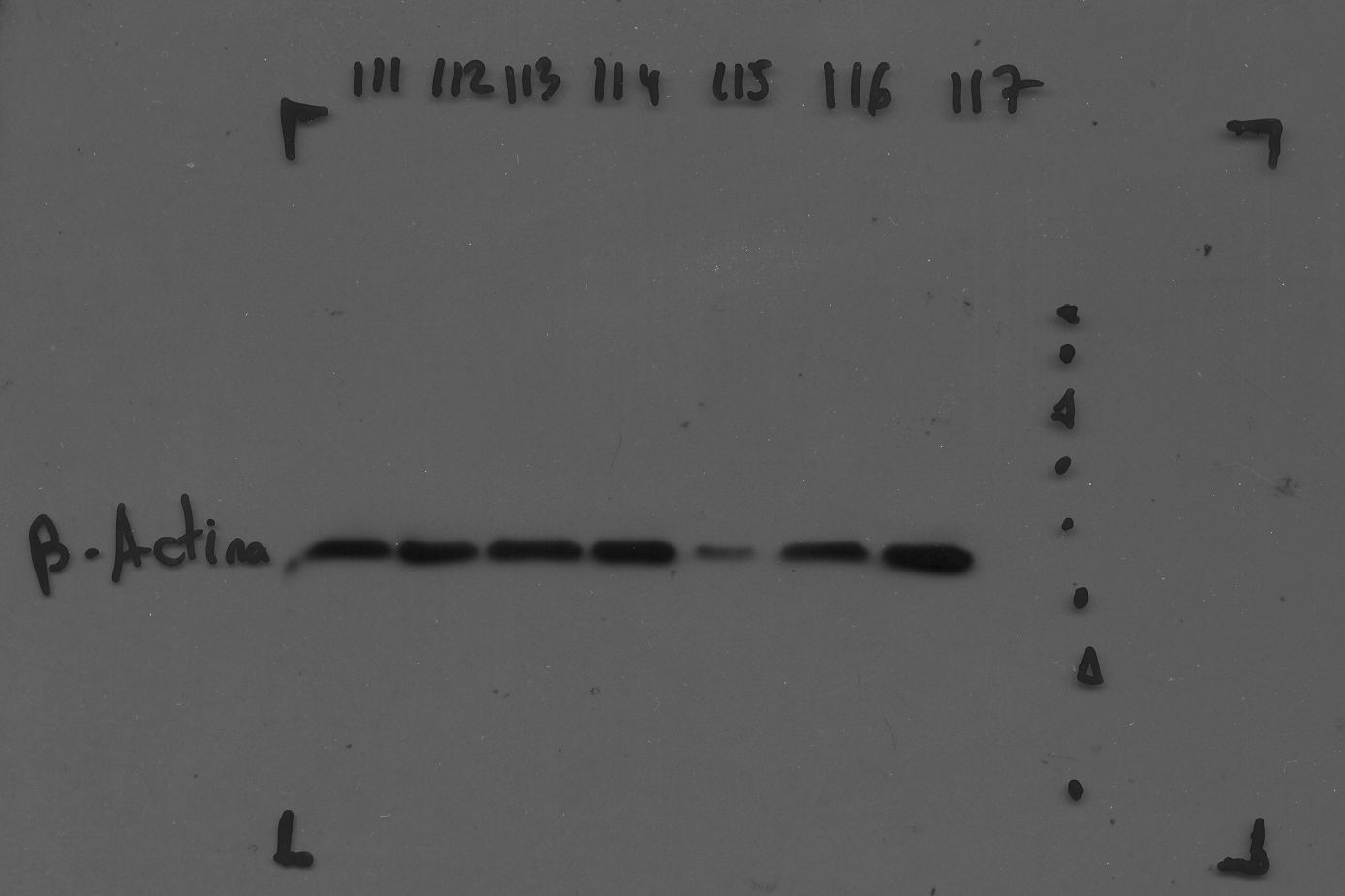

Supplement: Supplementary file 1 [file ijms-24-06453-s001.zip › Data + WBs/WBs/PBMCs/Fig. 3A/WB-b-Actin/WB-b-Actin:1.png]

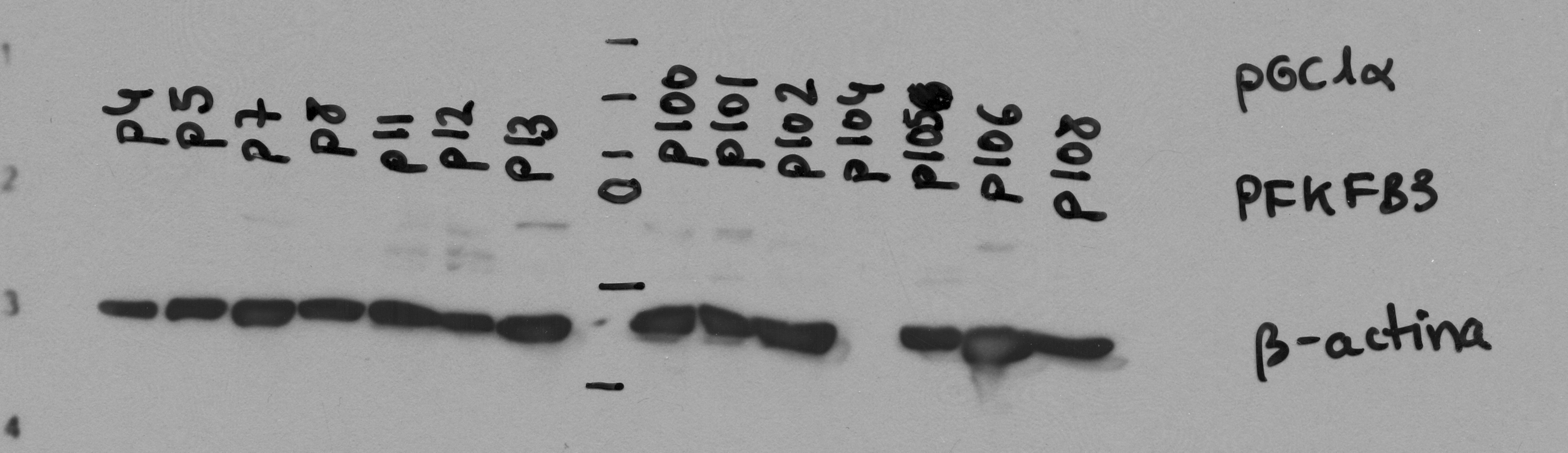

Supplement: Supplementary file 1 [file ijms-24-06453-s001.zip › Data + WBs/WBs/PBMCs/Fig. 3A/WB-b-Actin/WB-b-Actin:4.tiff]

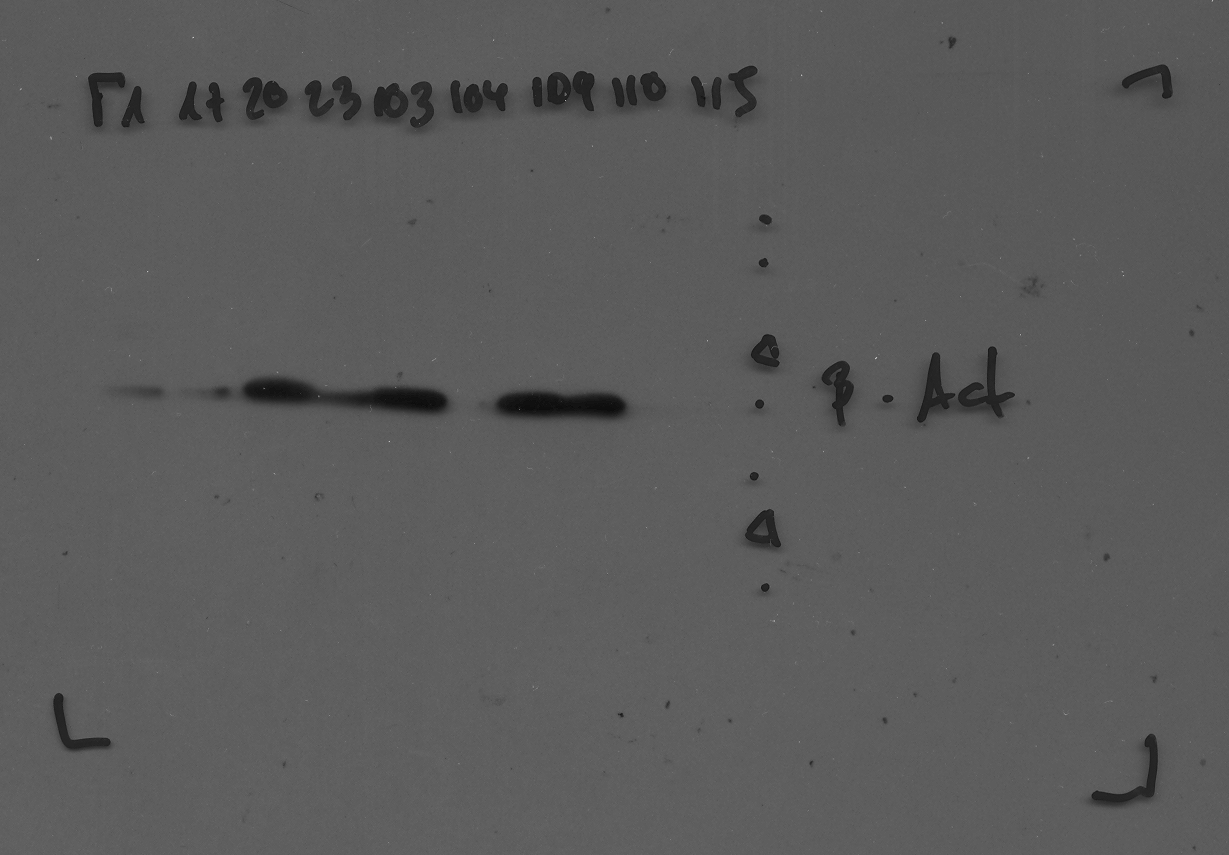

Supplement: Supplementary file 1 [file ijms-24-06453-s001.zip › Data + WBs/WBs/PBMCs/Fig. 3A/WB-b-Actin/WB-b-Actin:2.png]

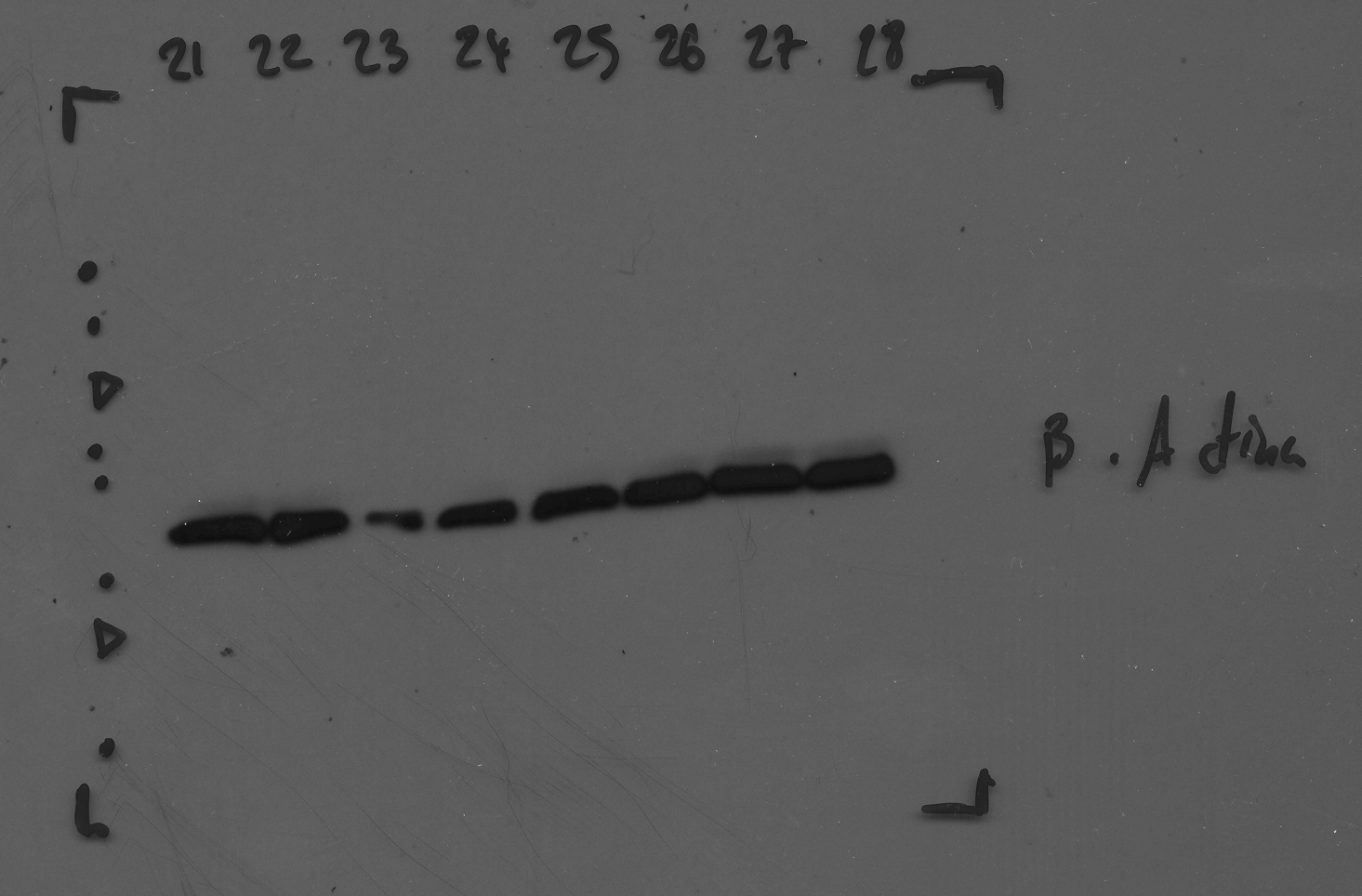

Supplement: Supplementary file 1 [file ijms-24-06453-s001.zip › Data + WBs/WBs/PBMCs/Fig. 3A/WB-b-Actin/WB-b-Actin:3.png]

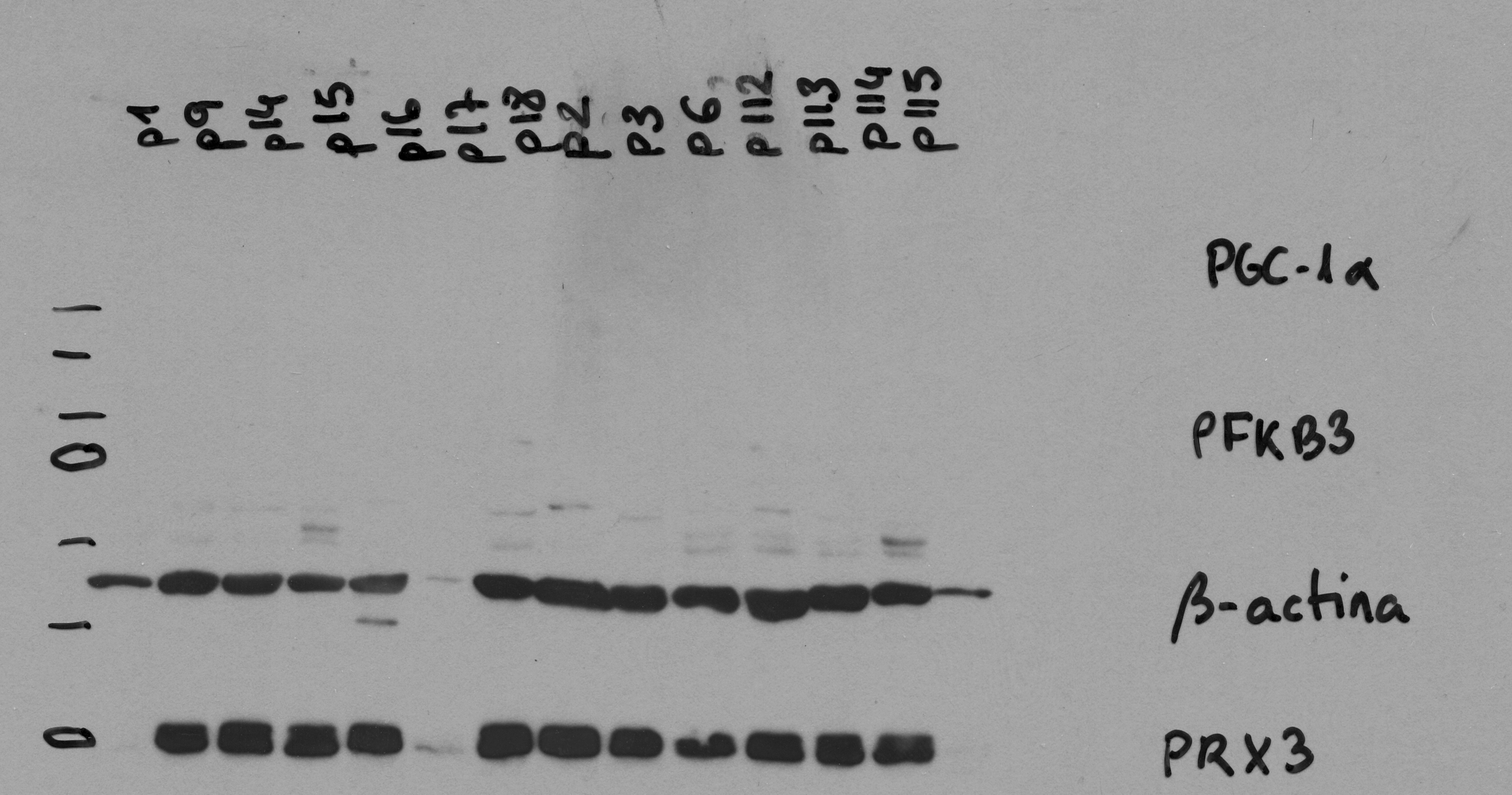

Supplement: Supplementary file 1 [file ijms-24-06453-s001.zip › Data + WBs/WBs/PBMCs/Fig. 3A/WB-b-Actin/WB-b-Actin:5.tiff]

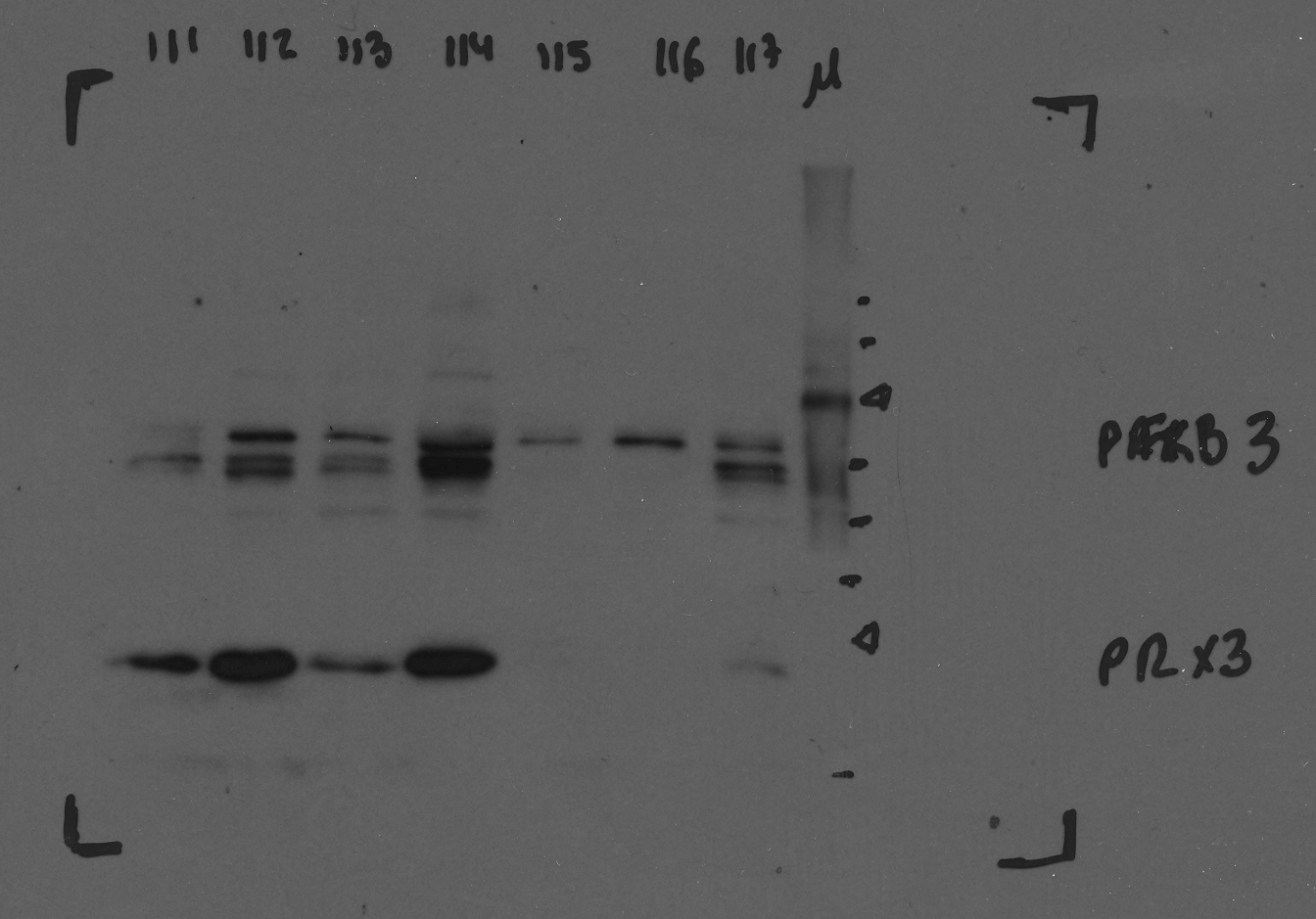

Supplement: Supplementary file 1 [file ijms-24-06453-s001.zip › Data + WBs/WBs/PBMCs/Fig. 3A/WB PFKFB3:PRX3/WB-PFKFB3-PRX3:1.png]

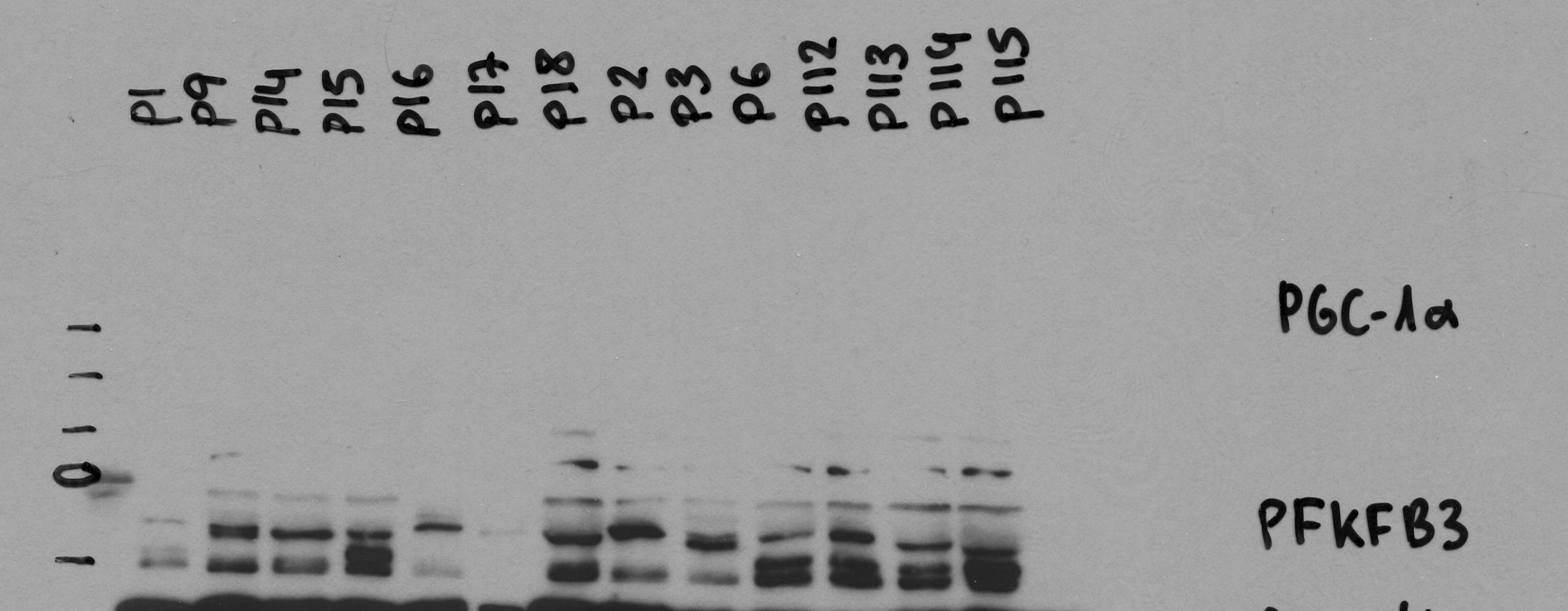

Supplement: Supplementary file 1 [file ijms-24-06453-s001.zip › Data + WBs/WBs/PBMCs/Fig. 3A/WB PFKFB3:PRX3/WB-PFKFB3:5.tiff]

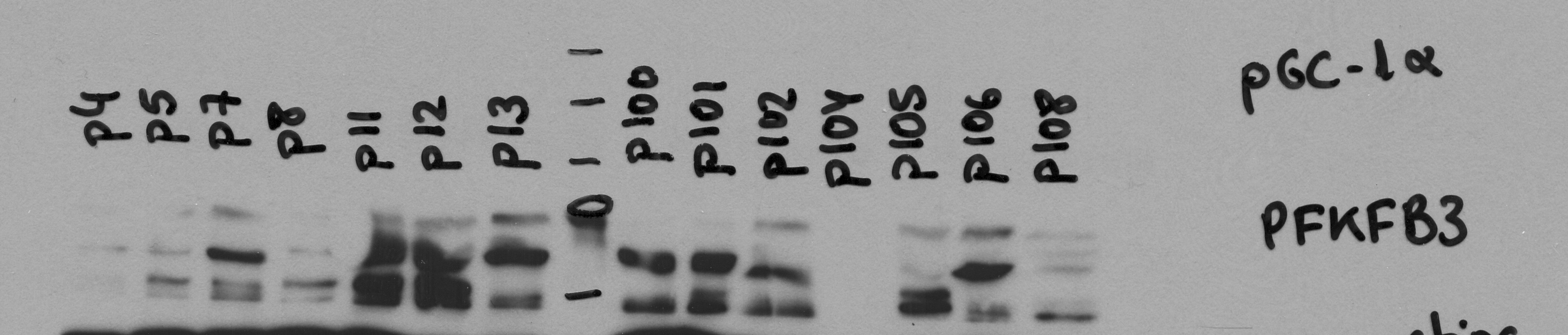

Supplement: Supplementary file 1 [file ijms-24-06453-s001.zip › Data + WBs/WBs/PBMCs/Fig. 3A/WB PFKFB3:PRX3/WB-PFKFB3:4.tiff]

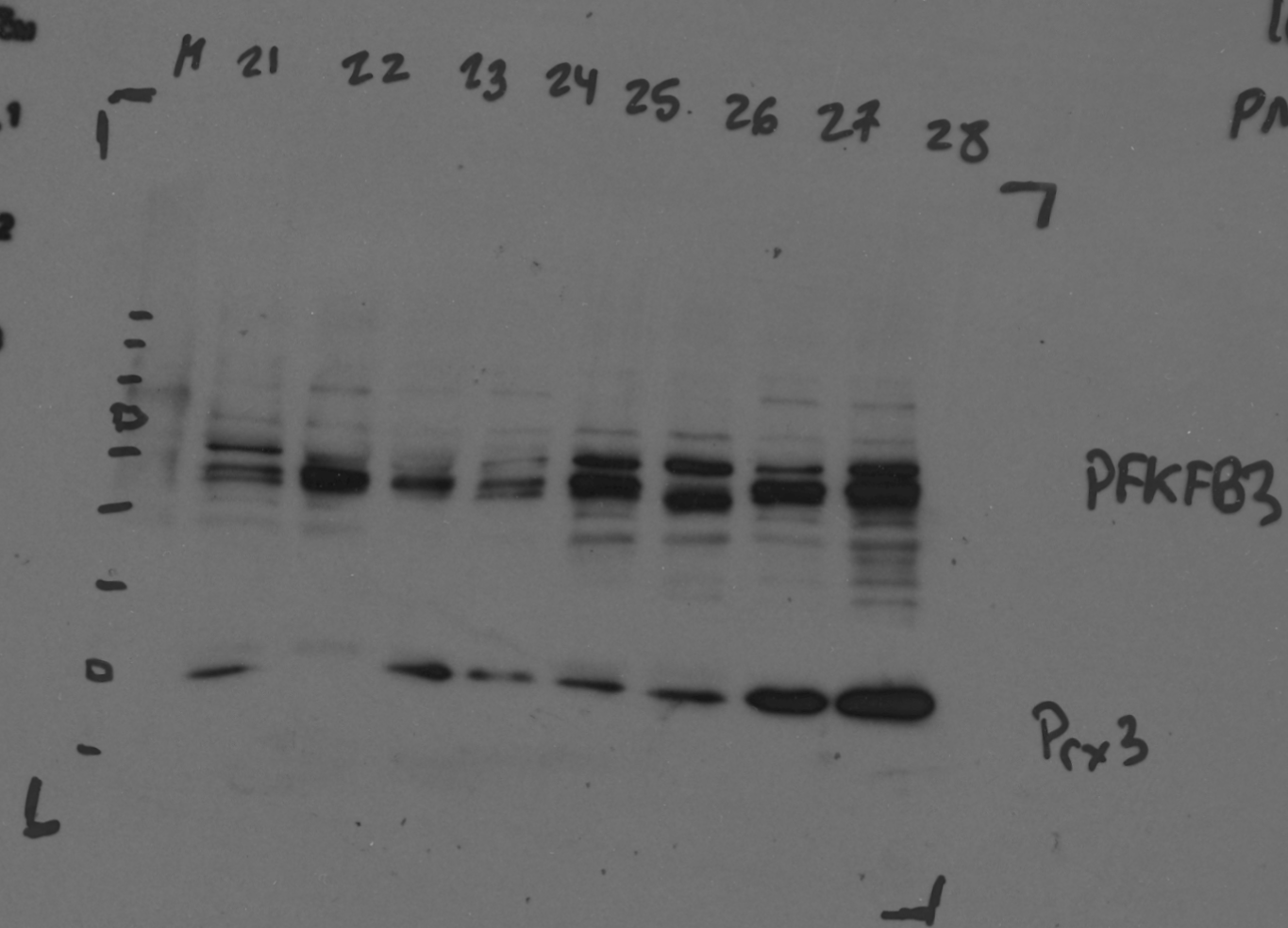

Supplement: Supplementary file 1 [file ijms-24-06453-s001.zip › Data + WBs/WBs/PBMCs/Fig. 3A/WB PFKFB3:PRX3/WB-PFKFB3-PRX3:3.tiff]

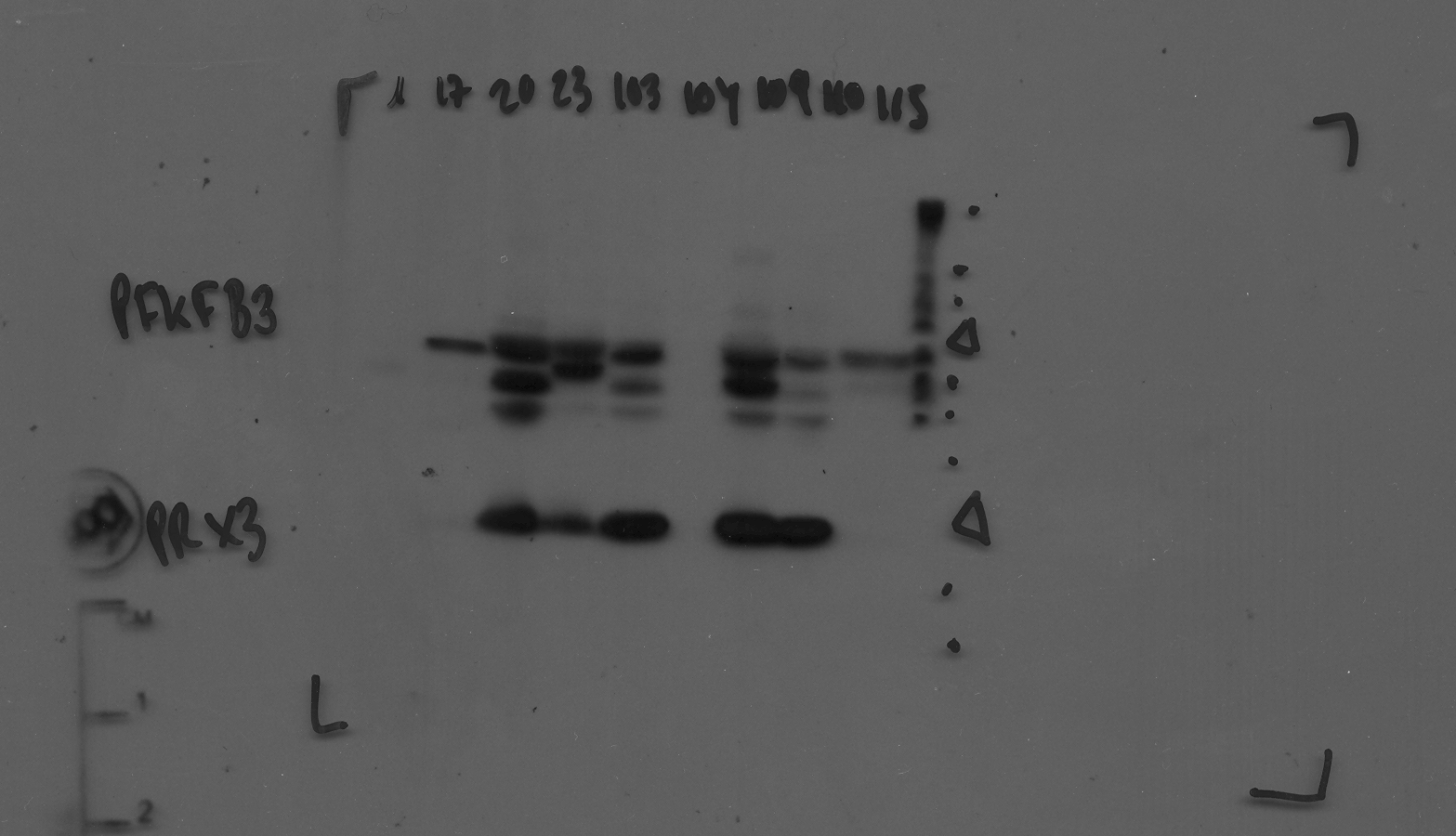

Supplement: Supplementary file 1 [file ijms-24-06453-s001.zip › Data + WBs/WBs/PBMCs/Fig. 3A/WB PFKFB3:PRX3/WB-PFKFB3-PRX3:2.tiff]

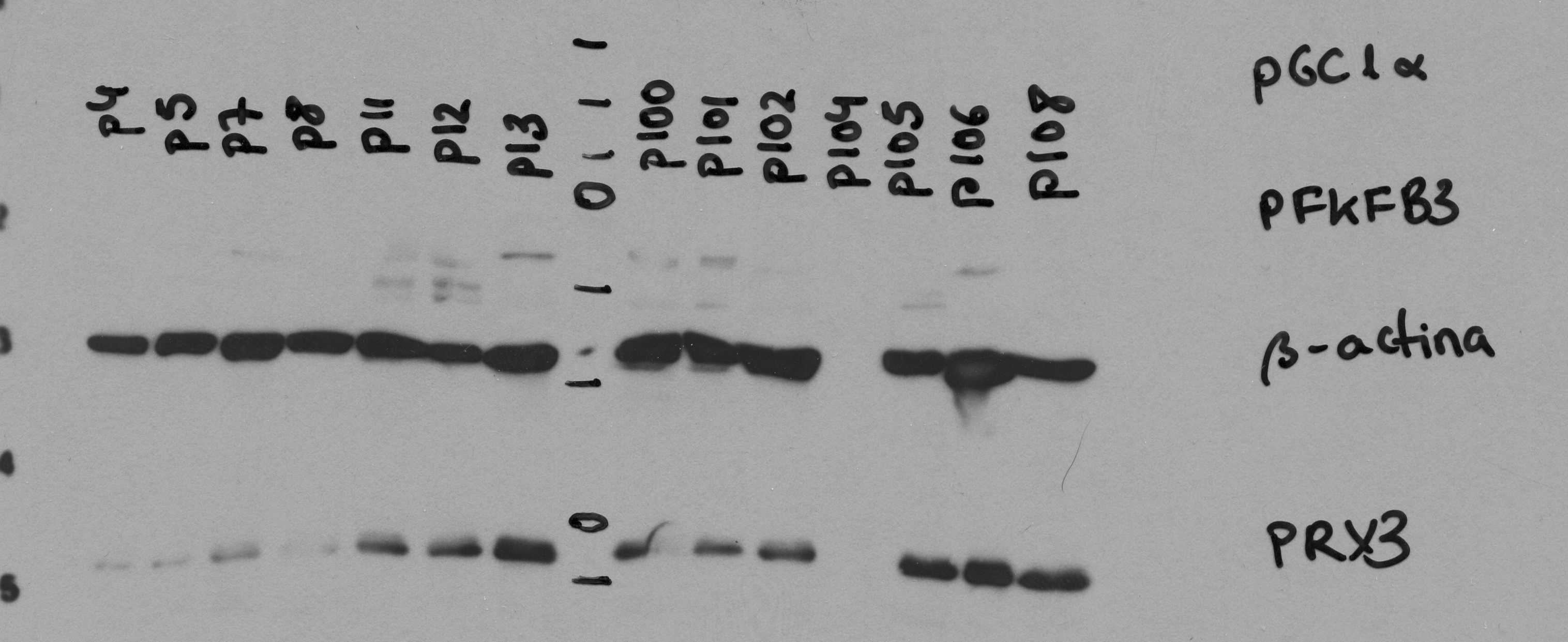

Supplement: Supplementary file 1 [file ijms-24-06453-s001.zip › Data + WBs/WBs/PBMCs/Fig. 3A/WB PFKFB3:PRX3/WB-PRX3:4.tiff]

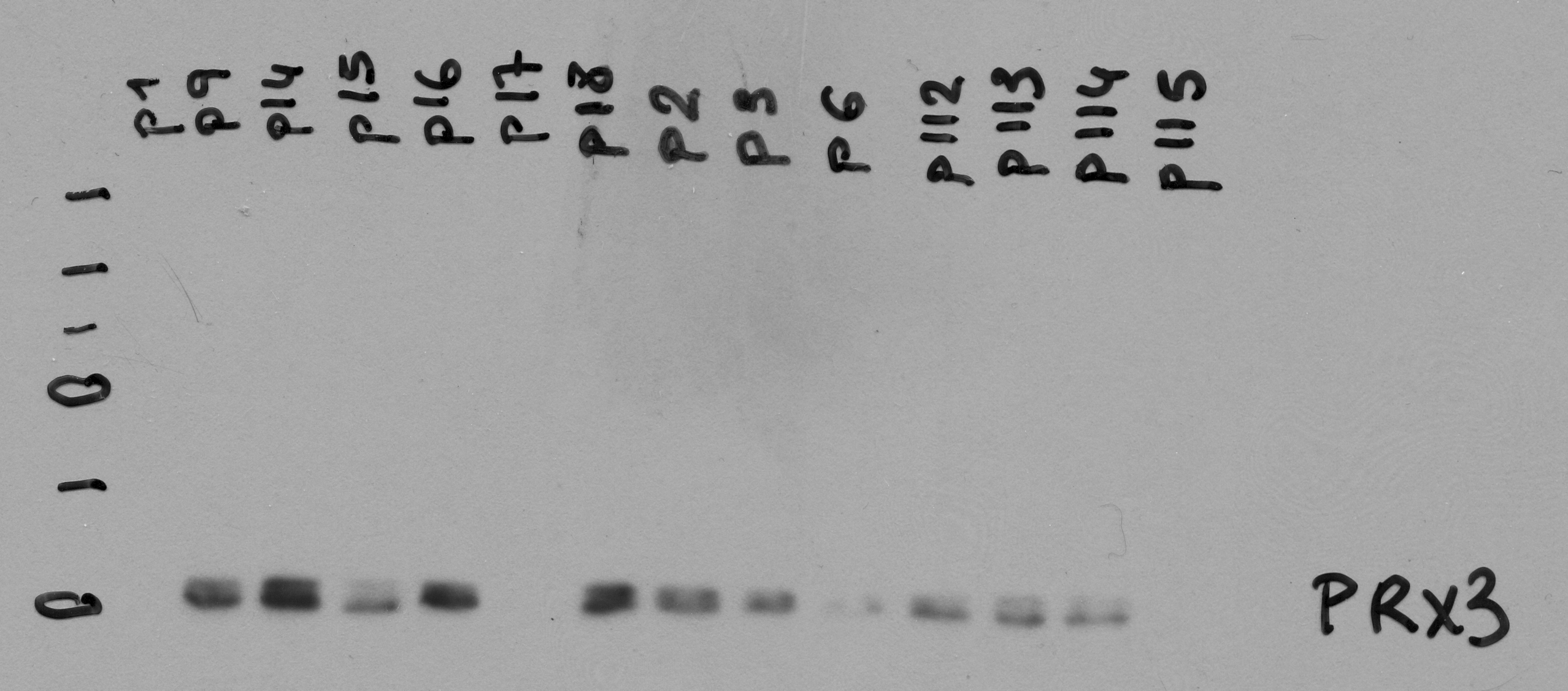

Supplement: Supplementary file 1 [file ijms-24-06453-s001.zip › Data + WBs/WBs/PBMCs/Fig. 3A/WB PFKFB3:PRX3/WB-PRX3:5.tiff]
